# Small intestinal mucosa-associated lymphoid tissue lymphoma with deep ulcer and severe stenosis: A case report

**DOI:** 10.1016/j.ijscr.2021.106539

**Published:** 2021-10-22

**Authors:** Kosuke Yoshimura, Hiroki Ohge, Shinnosuke Uegami, Yusuke Watadani, Toshinori Hirano, Shinya Takahashi

**Affiliations:** Department of Surgery, Graduate School of Biomedical and Health Sciences, Hiroshima University, 1-2-3 Kasumi, Minami-Ku, Hiroshima City, Hiroshima Prefecture 734-8551, Japan

**Keywords:** CT, computed tomography, DBE, double-balloon endoscopy, MALT, mucosa-associated lymphoid tissue, Mucosa-associated lymphoid tissue lymphoma, Small intestine, Tissue eosinophilia, Case report, Gastrointestinal tract

## Abstract

**Introduction:**

Although eosinophils are commonly present on the mucosa of the gastrointestinal tract, various pathological conditions may cause a secondary increase in eosinophil quantity.

**Presentation of case:**

A 78-year-old man was referred to our hospital due to abdominal pain. Examinations revealed an ulcerative lesion with white moss in the terminal ileum and severe stenosis on the oral and anal sides. Tissue biopsies obtained from the ulcer margins showed a predominance of chronic inflammatory cells and abundant eosinophils in addition to lymphocytes/plasma cells. Secondary causes of tissue eosinophilia were suspected; however, the diagnosis could not be confirmed because of atypical endoscopic findings. Partial resection of the ileum was performed for therapeutic and diagnostic purposes. Histopathology of the resected specimen identified a lymphoepithelial lesion with an invasive tendency. While CD20 staining was positive, MUM-1 and Bcl-6 staining were negative. Based on these findings, the lesion was diagnosed as a small intestinal mucosa-associated lymphoid tissue lymphoma (Lugano staging, stage II_1_).

**Discussion:**

Hypereosinophilia in this lesion was suggested to be secondary to chronic inflammation due to tumor growth or impaired transit.

**Conclusion:**

There is a type of gastrointestinal MALT lymphoma showing an invasive tendency. In such cases, it may demonstrate atypical findings and hypereosinophilia in gastrointestinal tissues.

## Highlights

• Eosinophils are commonly present on the GI mucosa floor.
• Abnormal/pathological conditions, include malignant lymphoma, can increase this concentration.
• Some gastrointestinal MALT lymphomas have a strong invasive tendency.
• Lymphocytes may infiltrate the deep ulcer and form severe stenosis.
• Sometimes, it is difficult to identify by endoscopic findings and needs surgical specimen for definitive diagnosis.

## 1 Introduction

Although eosinophils are often indigenous to the mucosa of the gastrointestinal tract, various pathological conditions may cause a secondary increase in eosinophil numbers on the gastrointestinal floor. Malignant lymphoma arising from the small intestine may be challenging to diagnose due to limited examination conditions; however, it could be diagnosed by histopathological examination of surgical specimens. This case report discusses a case of mucosa-associated lymphoid tissue (MALT) lymphoma of the ileum diagnosed by examining a surgical specimen from an eosinophil-rich deep ulcerative lesion and severe stenoses due to strong infiltrative tendency. This work has been reported in accordance with the SCARE criteria [1].

## 2 Presentation of the case

**Fig. 1:**
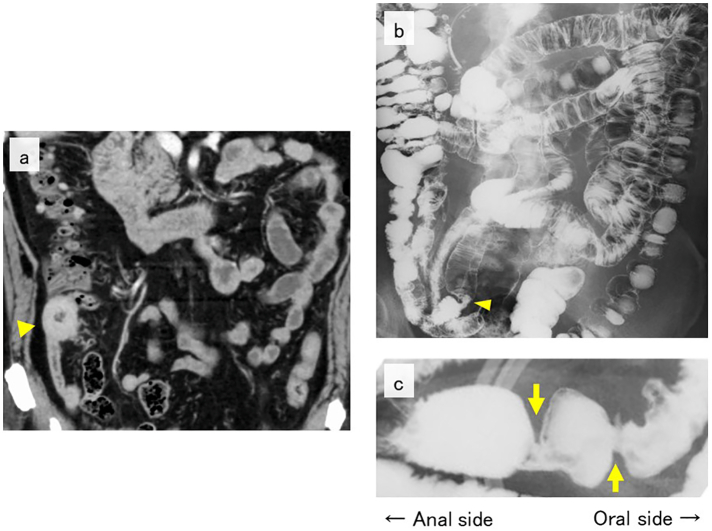
Radiological examinations. Contrast-enhanced CT shows circumferential wall thickening in the terminal ileum (a, arrowhead). Enterography shows a stenotic lesion 30–35 cm on the oral side from the terminal ileum (b, arrowhead), but no obstruction was observed in the entire gastrointestinal tract. Multiple stenotic lesions are shown (c, arrows); however, gastrografin could still pass through.

**Fig. 2:**
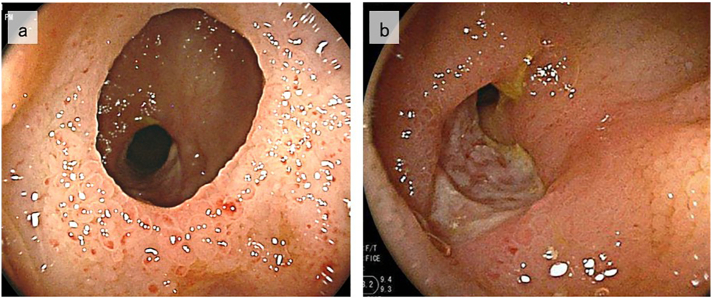
Small intestinal observation by colonoscopy. Colonoscopy shows a severe stenosis 30 cm towards the oral side from the terminal ileum (a) and another stenosis with a shallow depressed ulcerative lesion on the oral side (b).

**Fig. 3:**
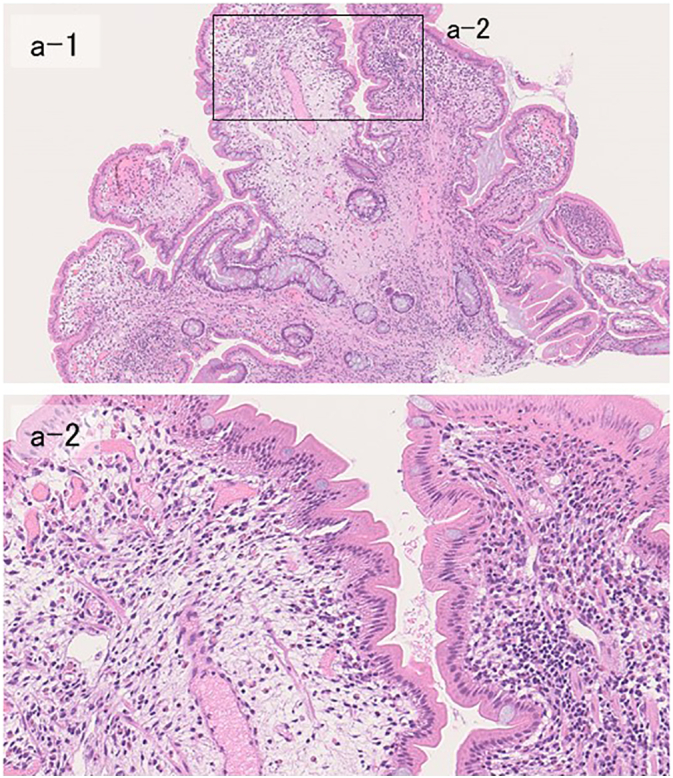
Histopathological findings of biopsy (H&E staining). The lesion comprises mainly chronic inflammatory cells (a-1; ×4). Magnification shows abundant eosinophils, lymphocytes, and plasma cells (a-2; ×40).

A 78-year-old man was referred to our hospital because of abdominal pain after eating. The patient had been on a diet and medications (bezafibrate, febuxostat, and tamsulosin hydrochloride) for hypertension, hyperlipidemia, hyperuricemia, and benign prostatic hyperplasia. There was no medical history of regular steroid or non-steroidal anti-inflammatory drug use. Laboratory results showed anemia (hemoglobin, 9.5 g/dL; hematocrit, 32.8%; iron, 14 μg/dL; and ferritin, 15.4 μg/dL). No other abnormalities were observed (white blood cell, 6570/μL; neutrophil, 60%; lymphocyte, 31%; monocyte, 6.8%; eosinophil, 2.0%; albumin, 4.2 g/dL; and lactate dehydrogenase, 163 IU/L). Tumor markers were within the reference ranges (CEA, 1.4 ng/mL; CA19–9, 11.6 U/mL; IL-2 receptor, 288 U/mL). Abdominal contrast-enhanced computed tomography (CT) showed a contrast-effective circumferential wall thickening at the terminal ileum (Fig. 1a). Enterography of the small intestine (transoral approach) revealed two stenotic lesions 30–35 cm away from the oral side of the terminal ileum (Fig. 1b, c). Double-balloon endoscopy (DBE) (transanal approach) revealed severe stenosis approximately 30 cm away from the oral side of the terminal ileum (Fig. 2a). Another stenosis with a shallow depressed ulcerative lesion was found on the oral side (Fig. 2b). However, the scope could not pass through the anal side stenosis, making detailed observation difficult. Tissue biopsies obtained from the ulcer margins showed lymphocytes, plasma cells, and eosinophils, which were predominantly chronic inflammatory cells (Fig. 3a-1, a-2). A deoxyribonucleic acid-polymerase chain reaction test for *Mycobacterium tuberculosis* using small intestinal tissues was negative. Capsule endoscopy was scheduled but could not be performed because the patent capsule could not pass through the stenotic lesions. We suspected the condition to be among diseases causing secondary hypereosinophilia, such as ischemic enteritis, drug-induced enteritis, malignancy, or inflammatory bowel disease, as well as eosinophilic enterocolitis (primary hypereosinophilia). However, the endoscopic findings of this lesion did not correlate with the results of tissue biopsy performed under limited conditions, making it challenging to confirm the diagnosis. As the stenotic lesions were severe, we planned partial ileal resection for therapeutic and diagnostic purposes.

The operation was performed under general anesthesia in the supine position. After laparotomy, we checked the entire small intestine and found no abnormalities other than these stenotic lesions, which were 30–35 cm away from the oral side of the terminal ileum. The ileum was anastomosed end-to-end after partial resection of a 10 cm ileal section that harbored the stenotic lesions. The anastomosis was located 25 cm away from the oral side of the terminal ileum.

**Fig. 4:**
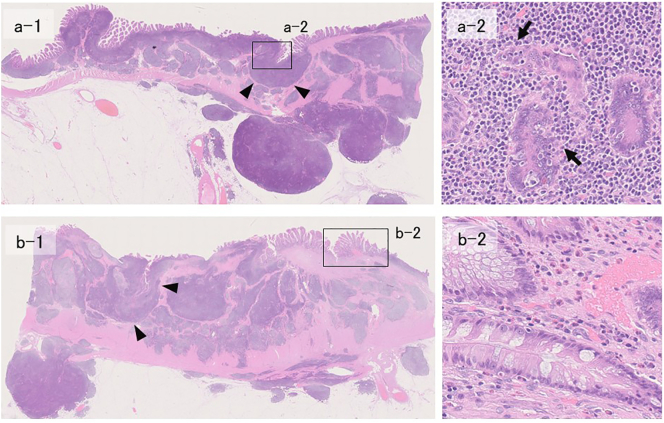
Histopathological findings of the surgical specimen (H&E staining) The tumor has invaded the mucosa, and the depressed ulcer is shown to have formed on the mucosal surface (a-1, b-1, arrowheads; ×4). On magnification, a lymphoepithelial lesion is observed in which small-to-medium-sized lymphocytes with inconspicuous nucleoli congregate and infiltrate existing gland ducts (a-2, arrows; ×100). Eosinophils accumulated in the normal mucosa (b-2; ×100).

**Fig. 5:**
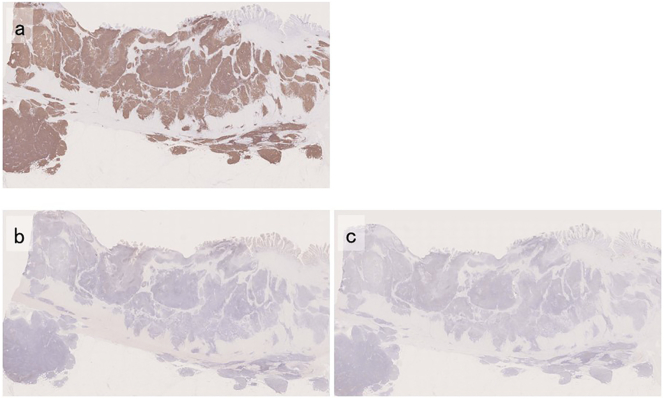
Histopathological findings of the surgical specimen (immunostaining) CD20 staining is diffusely positive (a; ×4), while MUM-1 staining (b; ×4) and Bcl-6 staining (c; ×4) are both negative.

Histopathological examination of the surgical specimen revealed that the tumor had invaded the intestinal mucosa and submucosa, and the caved-in ulcer had formed on the mucosa (Fig. 4a-1, b-1). On magnification, we found small-to-medium-sized aggregate lymphocytes with inconspicuous nucleoli infiltrating the existing glandular ducts. This finding is consistent with a lymphoepithelial lesion (LEL) (Fig. 4a-2). The normal mucosa present in some parts showed an accumulation of eosinophils (Fig. 4b-2). Immunohistochemical staining showed positive staining for CD20, suggesting that the tumor was composed mainly of B cells (Fig. 5a). In contrast, both MUM-1 staining (Fig. 5b) and Bcl-6 staining (Fig. 5c) were negative. Based on these findings, the final diagnosis was small intestinal MALT lymphoma (Lugano staging, stage II_1_).

Postoperative course was good, and the patient was discharged from our hospital on postoperative day 11. Esophagogastroduodenoscopy, capsule endoscopy, and positron emission tomography-CT were performed to examine the entire body. These examinations did not find any localized remnants or lesions in other organs. Based on this, no additional treatment was considered necessary, apart from continual close observation of the patient. One year and 7 months postoperatively, there is no sign of recurrence.

## 3 Discussion

Generally, eosinophils are spread throughout the mucous membranes of the gastrointestinal tract. However, when the gastrointestinal mucosal epithelium is damaged by food-derived antigens, pathogens, or other molecules, eosinophils accumulate in the mucosa. Various specific receptors, such as cytokines, chemokines, chemical mediators, and immunoglobulins, are expressed on the cell surface of eosinophils. The binding of molecules to these receptors triggers functions such as tissue damage, promoting and alleviating inflammation, tissue remodeling, immune modulation, and innate immunity [2]. This accumulation and functional induction of eosinophils in the gastrointestinal mucosa could be “primary” or “secondary” to inducing factors. In clinical practice, it is necessary to identify the causes behind the increased eosinophil count as it is often secondary to inflammatory bowel diseases, radiation, drugs, collagen diseases, vasculitis, fungal and parasitic infections, and neoplasms [3]. In cases where it is triggered by neoplasms, IL-5, a growth factor selective for the eosinophil lineage [4], as well as various cytokines such as IL-3 and GM-CSF, are produced by the tumors. These induce the accumulation and functional expression of eosinophils and affect the activation of other immune cells [2]. In the gastrointestinal tract, the production of cytokines by this process tends to be more common in inflammatory fibroid polyps and lymphomas than in other cancers [3]. Contrarily, eosinophilic enteritis is a disease of primary hypereosinophilia in gastrointestinal tissues. However, it can only be diagnosed after completely excluding diseases in which eosinophils are secondarily increased. Laboratory results or examination findings such as eosinophils in plasma, intestinal wall thickening on CT, and mucosal abnormalities on endoscopy are not absolute indicators [5].

MALT lymphoma is a rare type of B-cell lymphoma that is associated with abdominal pain. It accounts for only 8% of non-Hodgkin's lymphoma cases and occurs in extranodal sites throughout the body. The stomach is the most common primary site of origin (85%), whereas origination from the small intestine or colon is rare (8%–28%) [6]. Horie et al. reported only one case of MALT lymphoma out of 1328 cases in which DBE was performed [7]. Physical characteristics include abdominal pain as the main complaint, weight loss, fever, and night sweats. In cases where the MALT lymphoma originates from the small intestine, typical endoscopic findings are shallow ulcerative lesions. Histopathologically, most patients had mild pathogenesis and were considered to be in the localized stages. However, a type of disseminated disease that invades other organs has recently been suggested [8]. Therefore, it is best to diagnose the stage of the disease after examining the gastrointestinal tract and entire body.

In this present case, we considered the possibility that the eosinophils had increased secondary to comorbidities, included malignancy. However, it was challenging to consider primary small intestinal cancer or malignant lymphoma based on the endoscopic findings, i.e., coexistent deep ulceration and severe stenotic lesions. In addition, the patient did not meet the diagnostic criteria for eosinophilic enterocolitis. Histopathological examination of the resected specimen showed an invasive tendency, as it had invaded the mucosa and infiltrated lymphocytes accumulated under the mucosa. These features suggested that in addition to the formation of broad, shallow, and sunken ulcerative lesions on the mucosal surface, multiple severe stenotic lesions had formed due to disease progression. Hypereosinophilia in this lesion was suggested to be secondary to chronic inflammation due to tumor growth or impaired transit. In such cases, surgical resection is meaningful in terms of diagnosis and treatment.

## 4 Conclusion

There is a type of gastrointestinal MALT lymphoma showing a strong tendency to invade. In such cases, lymphocytes may infiltrate the mucosal surface at the base of the deep ulcer and form severe stenosis. In some cases that are difficult to identify by endoscopic findings, surgical specimen is required for definitive diagnosis.

## Sources of funding

This research did not receive any specific grant from funding agencies in the public, commercial, or not-for-profit sectors.

## Ethical approval

This is a case report and did not therefore require ethical approval from an ethics committee.

## Consent for publication

Written informed consent was obtained from the patient for the publication of this case report and accompanying images. A copy of written consent is available for review by the Editor-in-Chief of this journal upon request.

## CRediT authorship contribution statement

KY is the corresponding author. KY, HO, SU, YW, TH, and ST contributed to manuscript preparation. KY, HO, SU, and YW contributed to patient management. All authors read and approved the final manuscript.

## Registration of research studies

Not applicable.

## Guarantor

Shinya Takahashi, Department of Surgery, Graduate School of Biomedical and Health Sciences, Hiroshima University, Hiroshima, Japan.

## Declaration of competing interest

The authors declare no conflicts of interest.
